# Pharmacists’ Knowledge, Attitudes, Behaviors and Information Sources on Antibiotic Use and Resistance in Jordan

**DOI:** 10.3390/antibiotics11020175

**Published:** 2022-01-28

**Authors:** Ghaith M. Al-Taani, Sayer Al-Azzam, Reema A. Karasneh, Adel Shaban Sadeq, Nadia Al Mazrouei, Stuart E. Bond, Barbara R. Conway, Mamoon A. Aldeyab

**Affiliations:** 1 Department of Clinical Pharmacy and Pharmacy Practice, Faculty of Pharmacy, Yarmouk University, Irbid 21163, Jordan; g.altaani@yu.edu.jo; 2 Department of Clinical Pharmacy, Faculty of Pharmacy, Jordan University of Science and Technology, Irbid 22110, Jordan; salazzam@just.edu.jo; 3 Department of Basic Medical Sciences, Faculty of Medicine, Yarmouk University, Irbid 21163, Jordan; reema.karasneh@yu.edu.jo; 4 College of Pharmacy, Al Ain University, Al Ain 64141, United Arab Emirates; adel.sadeq@aau.ac.ae; 5 Department of Pharmacy Practice and Pharmacotherapeutics, College of Pharmacy, University of Sharjah, Sharjah 27272, United Arab Emirates; nalmazrouei@sharjah.ac.ae; 6 Pharmacy Department, Mid Yorkshire Hospitals NHS Trust, Wakefield WF1 4DG, UK; stuart.bond@nhs.net; 7 Department of Pharmacy, School of Applied Sciences, University of Huddersfield, Huddersfield HD1 3DH, UK; b.r.conway@hud.ac.uk; 8 Institute of Skin Integrity and Infection Prevention, University of Huddersfield, Huddersfield HD1 3DH, UK

**Keywords:** antimicrobial, pharmacist, resistance, knowledge, behavior, attitude, prescribing practices, antimicrobial stewardship

## Abstract

Antimicrobial resistance (AMR) is a serious healthcare problem that affects public health globally. Appropriate understanding and knowledge of prudent antimicrobial use and resistance, along with providing evidence-based information sources, are needed for informed antibiotic prescribing practices. This study aimed to assess the knowledge, opportunity, motivation, behavior of pharmacists and their information sources regarding antibiotic use and resistance in Jordan. An online cross-sectional questionnaire was developed and administered to pharmacists during the period of July–September 2021. The survey is an adapted version of the validated European Centre for Disease Prevention and Control (ECDC) survey for antibiotic use and resistance. Pharmacists from all sectors (*n* = 384), of whom 276 (71.9%) were community pharmacists, completed an online questionnaire. While respondents scored highly (>87%) on knowledge on effective use, unnecessary use, and associated side effects of antibiotics, lower scores were recorded for knowledge on the spread of antibiotic resistance (52.9%). Pharmacists support easy access to guidelines on managing infections in 56% of cases, and easy access to materials advising prudent antibiotic use and resistance in 39.8% of cases. One-third of respondents (37.0%) reported no knowledge of any initiatives on antibiotic awareness and resistance. Pharmacists were aware (13.3%), unaware (36.2%), or unsure (50.5%) of the existence of a national antibiotic resistance action plan. Pharmacists indicated an interest in receiving more information on resistance to antibiotics (55.2%), medical conditions for which antibiotics are used (53.1%), how to use antibiotics (45.1%), prescribing of antibiotics (34.4%), and links between the health of humans, animals, and the environment (28.6%). Findings can inform antimicrobial stewardship with required interventions to improve antibiotic use.

## 1. Introduction

Antimicrobial resistance (AMR) is a serious healthcare problem that affects public health globally, posing a significant challenge, and requires collaborative efforts to combat it [1,2]. AMR can complicate the care of patients and is implicated in increased hospitalizations, length of stay, increased costs, and increased morbidity and mortality [3,4,5]. It is postulated that as more antimicrobials are prescribed, so is the risk of AMR in primary and secondary sectors [1,6,7,8]. The association between antibiotic use and the development and spread of multidrug-resistant pathogens is well established [1,7,9,10]. It is important to increase public awareness of antimicrobial resistance to help develop means to fight this problem [11].

Antimicrobial stewardship should include a team of multidisciplinary healthcare professionals and is implemented to varying degrees in different countries [12,13]. Antimicrobial stewardship (AMS) has become an important emerging professional role for pharmacists [14,15]. Pharmacists can be involved at different stages in AMS, including patient evaluation, choice of antimicrobial, dispensing, and patient monitoring [14,15]. Antimicrobial stewardship has been shown to address the issue of AMR and promote the prudent use of antimicrobials and associated infection control strategies [16,17]. Nevertheless, appropriate understanding and knowledge of prudent antimicrobial use, and the spread of antimicrobial-resistant microorganisms are important drivers for the prevention and control of AMR [18,19,20]. Behavior change in favor of improving antibiotic use and resistance outcomes is an essential strategy; a framework to address this behavior change was devised by the European Centre for Disease Prevention and Control and was adopted by the present study [13,21]. This framework addressed capabilities, opportunities, and motivations that facilitate correct behavior (COM-B). Capability is related to the ability to carry out behavior, and motivation is about the desire to carry out such behavior, whereas opportunity is related to factors that make the behavior prompted [13,21]. Healthcare providers can make an intervention using any of the capabilities, opportunities, and motivations in order to modify behavior [21]. Pharmacists are engaging in newer roles as the profession becomes more patient centered, bearing in mind that pharmacists are the most accessible healthcare professionals, and the overwhelming majority of antimicrobial prescribing occurs in primary healthcare settings. Pharmacists are also involved in information provision to the public and other healthcare professionals and are ideally positioned to provide timely and tailored advice on prudent antimicrobial use and efforts to reduce antimicrobial resistance to the public and other healthcare professionals [22,23,24,25]. In order to provide appropriate health care, an evidence-based approach should be employed, and as such, it is important to rely on high-quality resources. This ethical obligation is threatened by information accessed on the internet with varying quality that may be employed in clinical care without prior scrutinization [26,27].

The present study aimed to evaluate pharmacists’ knowledge, attitudes, and behaviors about antibiotics, antibiotic use, and antibiotic resistance in Jordan. A secondary aim was to explore pharmacists’ preferred sources of information and evaluate their awareness of available information and initiatives on prudent antibiotic prescribing in Jordan. 

## 2. Results

A total of 384 respondent pharmacists constituted the study sample. There were more females (70.1%) than males, and most were within the age group of 22–35 years (78.6%). The predominant role was most frequently identified as generalist (313/384, 81.5%), and the most predominant practice places (under the generalist category) were the community pharmacy (276/384; 71.9%), hospital (38/384; 9.9%), and industry (12/384; 3.1%). Seventy percent of the respondents had been practicing in their role within the pharmacy profession for 5 years or less. The demographic and background variables are summarized in Table 1.

Respondents achieved high scores in assessment of actual knowledge related to items about antibiotic use (Table 2). Considerably lower scores were achieved on questions related to the increased risk of antimicrobial resistance in every person treated with antibiotics (69.0% answered correctly), the spread of resistance from one person to another (52.9%), and whether a person can carry antibiotic resistant bacteria (63.5%). A low score was observed when asked if it is legal to use antibiotics to stimulate the growth of farm animals (19.0%; Table 2). A summated score was calculated for each respondent, indicating the sum of the number of correct answers in the questions about actual knowledge. The average score for the study sample was estimated to be 5.7 (±1.33). Only 18 respondents (4.7%) answered all the questions correctly.

High levels of perceived knowledge were expressed by the study sample, e.g., 88.0% of the respondents strongly agreed or agreed with the statement concerning knowledge of antibiotic resistance (Table 3). On the assessment of the perceived knowledge regarding environmental factors related to antimicrobials, only 42.7% of respondents strongly agreed or agreed with the statement about the role of environmental factors as a contributor to antibiotic resistance. On the items detailing opportunities, 56.0% of the respondents strongly agreed or agreed with the statement about having easy access to guidelines that they need on managing infections, and 39.8% of the respondents strongly agreed or agreed with the statement about having easy access to materials they need to give advice on prudent antibiotic use and resistance. In relation to motivation, 68.0% of the respondents strongly agreed or agreed with the statement about the knowledge of a connection between dispensing antibiotics and emerging antibiotic resistance. Seventy percent of the respondents strongly agreed or agreed with the statement about having a key role in helping control antibiotic resistance (Table 3).

Regarding the behavior of pharmacists in promoting prudent antibiotic use, 48.2% of the respondents dispensed an antibiotic once a day or more than once a day in the previous week, 13.3% of the respondents gave out resources on prudent antibiotic use or management of infection once a day or more than once a day in the previous week, and 36.2% of respondents gave out advice related to prudent antibiotic use or management of infections once a day or more than once a day in the previous week. Details regarding these items are summarized in Table S1—Supplementary Material. A sub-analysis of the respondent community pharmacists was undertaken to examine the impact of age on the total knowledge score; however, the results were statistically insignificant (*p* = 0.070). On the other hand, when the association between the age of the community pharmacist respondents and those who answered all the knowledge questions correctly was conducted, the results revealed that pharmacists who were younger than 35 years old were more likely to answer all the questions correctly (*p* = 0.050).

Regarding information resources on antibiotic use and resistance, the most frequently (80.5%) used social media sites for professional activities were Facebook, followed by Instagram (20.1%), Google (18.5%), LinkedIn (11.7%) and YouTube (11.2%) (Table S2—Supplementary Material).

The survey assessed reasons why respondents were not able to give out advice or resources as they dispense antibiotics, and it was found that the most frequently cited reason (45.3%) was that the patient was not interested in information. Other common reasons were that there were no resources available (37.0%) and insufficient time (27.1%). Only 25.8% of respondents were able to give out advice or resources as needed. The most frequent information resource to which respondent pharmacists regularly referred in the management of infection was continuing education training courses (60.9%). Other frequent resources reported were clinical practice guidelines (35.9%), previous clinical experience (29.2%) and professional resources/publications (28.4%). In relation to having received any information about avoiding unnecessary dispensing of antibiotics in the last 12 months, 57.0% of the respondents answered yes, 35.4% answered no and 7.6% were unsure (Table S3—Supplementary Material).

**Figure 1:**
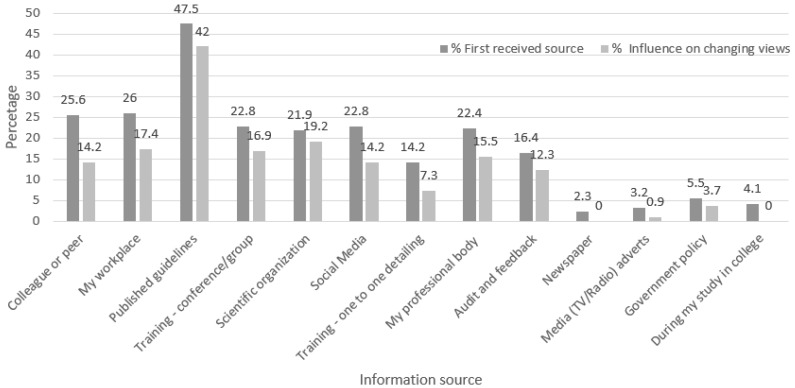
Sources of information that pharmacists first get about avoiding indiscriminate antibiotic use and their influence in changing pharmacists’ views.

A sub-analysis of those who answered yes to whether they received any information about avoiding unnecessary dispensing of antibiotics in the last 12 months was performed. Results revealed that the common resources used were published guidelines (47.5%), their workplace (26.0%) and colleague or peer (25.6%). The overwhelming majority (95.0%) of respondents believed that the information received contributed to changing their views about avoiding unnecessary dispensing of antibiotics. The most common influential resources on changing views were published guidelines (42.0%), scientific organizations (19.2%) and the pharmacist workplace (17.4%). Again, the majority (92.7%) of respondents believed that the information received had an impact on changing their practice in dispensing antibiotics. Few (3.2%) respondents believed that the information received did not have an impact in changing their practice in dispensing antibiotics; the reasons given were that they did not think that the message was important, they forgot, and they did not have the opportunity. Figure 1 compares access to sources of information pharmacists get firstly about avoiding indiscriminate antibiotic use and their influence in changing pharmacists’ views.

Regarding topics about which they would like to receive more information, 55.2% of respondents selected resistance to antibiotics, 53.1% selected medical conditions for which antibiotics are used, 45.1% chose how to use antibiotics, 34.4% chose prescription of antibiotics and 28.6% selected links between the health of humans, animals and the environment. Regarding initiatives in Jordan that focus on antibiotic awareness and resistance, the most common initiatives reported were awareness arising from professional organizations (24.0%), conferences/events focused on tackling antibiotic resistance (21.9%), TV or radio advertising for the public (18.2%) and toolkits and resources for healthcare workers (18.2%). Full details regarding awareness of these initiatives by the respondents is presented in Table 4. Regarding the item “Does your country have a national action plan on antimicrobial resistance” 50.5% of the respondents were unsure, 36.2% answered no and only 13.3% said yes.

## 3. Discussion

Antimicrobials have been the frontline in the human battle against microorganisms; however, excessive use is seriously threatening their efficacy [28]. Health professionals can have a pivotal role in addressing AMR, particularly those who deal most frequently with antibiotics. As with other professionals, pharmacists are involved in routine work with antimicrobial use [29]. Thus, it is important to identify and characterize barriers to prudent antimicrobial use, at the levels of capabilities, opportunities, and motivations. This will help in providing interventions designed to influence the behaviors of pharmacists toward better use of antibiotics, thereby mitigating antimicrobial resistance. Key findings of the present study highlighted that the respondent pharmacists had good actual and perceived knowledge regarding antibiotic use and resistance. Poorer knowledge regarding certain categories, such as the spread of resistance, the ability of patients to carry resistance microorganisms, and items related to the impact of antibiotics on the environment, was reported. There were some issues reported in items related to opportunity in this study, particularly when it comes to easy access to guidelines and patient education material. Pharmacists are generally well motivated. The results presented are the first to assess the knowledge, attitudes, and behaviors of pharmacists in the Middle East in antibiotic use and resistance, using an adapted version of the validated instrument of the ECDC [13]. Respondents were more frequently young, within the age group of younger than 35 years old, and it is anticipated that this age group can absorb and uptake novel initiatives, such as antimicrobial stewardship. Indeed, pharmacists more than 35 years old were shown in other studies as being less confident in promoting prudent antimicrobial use [30]. The present study found that pharmacists who were younger than 35 years old were more likely to answer all the knowledge questions correctly (*p* = 0.050). The study group consisted primarily of respondents who have been practicing in their current profession for 5 years or less (70%).

Overall, the responding pharmacists’ performance was good on knowledge of issues related to antibiotic use and resistance. This was apparent in the items related to, among others, antibiotic use (not effective) against viruses or cold and flu. On the contrary, performance on actual knowledge scales was considerably lower relating to the spread of resistance and the increased risk of resistance in every person treated with an antibiotic. The results are consistent with a study performed in 30 EU/EEA countries, which showed that 97% of the pharmacists surveyed agreed or strongly agreed to the statement “I know what antibiotic resistance is” [21]. Evidence supports that healthcare professionals, in general, can frequently identify the link between antibiotic use (prescribing, administration, and dispensing) and the evolution and transmission of antibiotic resistance [31,32,33,34,35,36,37]. High perceived knowledge was expressed by the respondent pharmacists. They reported high knowledge in what antibiotic resistance is, what information to give out to patients and how to use antibiotics. This was consistent with recent survey data of Hungarian community pharmacists who reported having appropriate knowledge in relation to antimicrobial use [30]. Regarding responses in relation to environmental factors associated with antibiotics, pharmacists demonstrated low knowledge, highlighting the need to improve it.

Knowledge is not the sole factor that influences healthcare providers’ and pharmacists’ behavior toward more prudent antibiotic use [18,38,39]. Having the opportunity and the motivation, in conjunction with appropriate knowledge, to address indiscriminate antimicrobial use is important for pharmacists to deliver appropriate antibiotic use practices. Relatively lower percentages were observed in this study for opportunity items such as access to guidelines and materials related to antibiotic use. These findings highlight an area for improvement for pharmacists in Jordan. Regarding motivation, pharmacists were aware of the connection between dispensing an antibiotic and resistance outcomes and that they play a key role in controlling antibiotic resistance. This is consistent with other studies [32].

In this study, almost half of the respondents dispensed antibiotics. However, fewer pharmacists provided advice or resource to patients once a day or more to support prudent antimicrobial use. It is well known that indiscriminate antibiotic use is associated with problems in the healthcare systems [1,7]. Pharmacists should provide advice in order to mitigate AMR risks. In order for pharmacists to provide appropriate advice to their patients, they should have the appropriate knowledge of issues concerning antibiotic use and resistance and be able to refer to professional drug information resources for appropriate patient care.

Bearing in mind that antimicrobial stewardship is not applied routinely in the community setting, efforts from community pharmacists should focus on audit, monitoring and education [25]. In the present study, the most common reasons for not giving out advice to patients regarding prudent antibiotic use were patient non-interest in information and lack of available resources. It has been reported that a considerable percentage of community pharmacists support the importance of patient education in the reduction of infectious diseases [27]. Respondent pharmacists relied most commonly on continuous education courses and clinical practice guidelines that are used routinely in clinical practice. Published guidelines are key in improving the prescription of antibiotics [40,41,42]. Respondent pharmacists sought information most commonly from published guidelines, and this resource has had the most influence on changing their view. Interestingly, social media was an initial source of information for a considerable number of respondent pharmacists (22.8%). However, this source of information had low influence on changing their view. Of note, social media comes with its problems in quality of information and associated risks [43,44]. Most respondents were not aware of the national action plan on antimicrobial resistance in Jordan [45]. Professional organizations and conferences/events focused on tackling antibiotic resistance were identified as one of the main initiatives which focus on antibiotic awareness and resistance. These results highlight the need for increased engagement of pharmacists in educational interventions that focus on prudent antibiotic use. In Italy, this need was identified by pharmacists themselves in a focused survey [29]. Furthermore, in Russia, the most common source of drug information was training sessions [46].

The present study has the strength of using well developed and validated methods [13,21]. Nevertheless, it has some limitations. The study can be limited by social desirability bias, manifesting as reporting good behavior. This bias was controlled to a degree by anonymous distribution of the survey. Online distribution might exclude those who do not use internet and/or smart phones. Such bias is not expected to be significant, as it was reported that the literate population, including healthcare professionals, frequently uses the internet and smart phones [47]. It was noted that more female pharmacists responded to the survey. This indicates representativeness of the sample, as there are more female pharmacists registered with the Jordan Pharmacists Association, the professional body in Jordan. Higher responses from females were also reported in other recent surveys [21,29]. In the present study, there was no means to confirm that the respondents were pharmacists, as the survey completion was self-completed by the participants. Using this online distribution, it is possible that those who are interested in antimicrobial resistance are more likely to respond.

In conclusion, the present study assessed the knowledge, opportunity, motivation, behavior and information sources of pharmacists in Jordan regarding the antibiotic use and resistance. The results of this study highlight the need for public health interventions (e.g., educational or communication campaigns). Efforts are needed to improve the awareness of pharmacists about appropriate antibiotic use and resistance. The findings can inform antimicrobial stewardship with required interventions to improve antibiotic use. Policymakers, clinicians, and professional bodies can be informed about the status quo of the pharmacists in their progress toward better behavior to support prudent antimicrobial use via the model capabilities, opportunities and motivators in the present study.

## 4. Materials and Methods

A 10 min online structured cross-sectional survey was developed and distributed to pharmacists in Jordan (Supplementary File S1). The survey was uploaded on a web-based survey software, Google Forms, for data collection. The inclusion criteria for the respondents were holding a bachelor’s degree in pharmacy or having a higher educational qualification.

An invitation to participate in the study reached the target population online, using the social media platforms of pharmacists’ professional groups, e.g., Facebook. The invitation included a brief description of the study and a link to the survey. Participants were informed that the survey was anonymous, i.e., no personal identifiers of respondents or geolocations were collected, and that the responses were confidential. The completion of the questionnaire was voluntary, and participants could withdraw from the study at any time. The survey was administered to pharmacists during the period of July–September 2021.

The present study was based on an adapted version of the carefully developed and thoroughly validated survey by the European Centre for Disease Prevention and Control (ECDC) [13,21]. Adaptations included targeting only pharmacists instead of all healthcare workers and students, addition of the region in Jordan instead of the country, relating this question to Jordan “The use of antibiotics to stimulate growth in farm animals is legal in Jordan”, and including only the choices of available sources of information to pharmacists in Jordan. The adapted survey was subject for review, by the research team, to ascertain the face and content validity. A pilot distribution of the developed questionnaire was carried out by 10 pharmacists online, and the pilot data were excluded from the final analysis. Based on the experts’ review and the pilot distribution, a final version of the survey was confirmed. The survey was available to the respondents in Arabic and English languages to choose from as they preferred.

Based on the COM-B model, the survey was composed of the following parts. Demographic and background items (5 items) included sex, age, experience, governate and predominant role. Items on capability, included items related to quantification of the degree of knowledge and level of understanding of the issue of AMR. They included assessment of both actual knowledge and perceived knowledge. In the assessment of the actual knowledge of antimicrobial resistance (8 items), the respondents choose whether the key knowledge statement is true or false. Assessment of perceived knowledge (5 items) was performed using a Likert scale. Three items on opportunity used a Likert scale that assessed access to guidelines and educational material. Two items on motivation included a Likert scale that assessed how much the respondents agree with the implication of the dispensing of antibiotics and the emergence of resistance and the important role of the pharmacist in controlling antibiotic resistance. Three items on behavior included how often the pharmacist dispenses antimicrobials and provides advice for the individual patients. Other items related to information sources for the pharmacist regarding the antimicrobial use and resistance as well as initiatives at the country level that focus on antibiotic use and resistance.

### Data Analysis

The sample size was determined, using the Raosoft sample size calculator (http://www.raosoft.com/samplesize.html; last accessed: 1 June 2021), based on a margin of error of 5%, confidence level of 95%, population size of 25,700 registered pharmacists with the Jordan Pharmaceutical Association [48] and a response distribution of 50%. The calculated sample size was 379.

Descriptive statistics, such as means, frequencies and standard deviations, were used to summarize the data. Means were compared using independent samples t-test and one-way ANOVA test. The chi square test was used to assess the association between categorical variables. A *p*-value of less than 0.05 was considered statistically significant. The Statistical Package for Social Sciences (SPSS^®^ version 26) was used to run the analyses.

## Figures and Tables

**Table 1:** Demographic and background variables.

Variables		*n*	%
Gender	Female	269	70.1
	Male	115	29.9
Age	22–25 years	164	42.7
	26–35 years	138	35.9
	36–45 years	57	14.8
	46–55 years	21	5.5
	56–65 years	4	1
Region	Middle	271	70.6
	North	85	22.1
	South	28	7.3
Predominant role (50% of your time)	Academia/Research	31	8.1
	Generalist (e.g., community pharmacy)	313	81.5
	Specialist	40	10.4
How many years have you been practicing in your current profession?	5 years or less	270	70.3
	6–15 years	81	21.1
	16 years or more	33	8.6

**Table 2:** Actual knowledge of the respondents in relation to antibiotic use and resistance.

Key Knowledge Questions	Correct Answer	Answer	*n*	%
Antibiotics are effective against viruses	False	False	345	89.8
		True	26	6.8
		Unsure	13	3.4
Antibiotics are effective against cold and flu	False	False	335	87.2
		True	42	10.9
		Unsure	7	1.8
Unnecessary use of antibiotics makes them become ineffective	True	False	20	5.2
		True	353	91.9
		Unsure	11	2.9
Taking antibiotics has associated side effects or risks such as diarrhea, colitis, allergies	True	False	16	4.2
		True	352	91.7
		Unsure	16	4.2
Every person treated with antibiotics is at an increased risk of antibiotic resistant infection	True	False	73	19.0
		True	265	69.0
		Unsure	46	12.0
Antibiotic resistant bacteria can spread from person to person	True	False	112	29.2
		True	203	52.9
		Unsure	69	18.0
Healthy people can carry antibiotic resistant bacteria	True	False	44	11.5
		True	244	63.5
		Unsure	96	25.0
The use of antibiotics to stimulate growth in farm animals is legal in Jordan	False	False	73	19.0
		True	40	10.4
		Unsure	271	70.6

**Table 3:** Perceived knowledge, opportunity and motivation of pharmacist regarding appropriate antibiotic use and resistance.

Item	SA	A	D	SD	N/A	U	IDU
Perceived knowledge							
I know what antibiotic resistance is	126	212	4	8	-	25	9
	32.8%	55.2%	1.0%	2.1%	-	6.5%	2.3%
I know what information to give to individuals about the prudent use of antibiotics and antibiotic resistance	76	229	13	4	9	49	4
	19.8%	59.6%	3.4%	1.0%	2.4%	12.8%	1.1%
I have sufficient knowledge about how to use antibiotics appropriately for my current practice	81	245	18	3	4	31	2
	21.1%	63.8%	4.7%	0.8%	1.1%	8.1%	0.5%
Environmental factors such as waste water in the environment are important in contributing to antibiotic resistance in bacteria from humans	26	138	43	13	-	130	44
	6.8%	35.9%	11.2%	3.4%	-	31.2%	11.5%
Excessive use of antibiotics in livestock and food production is important in contributing to antibiotic resistance in bacteria from humans	69	179	16	6	-	92	22
	18.0%	46.6%	4.2%	1.6%	-	24.0%	5.7%
Opportunity							
I have easy access to guidelines I need on managing infections	29	186	63	18	7	75	6
	7.6%	48.4%	16.4%	4.7%	1.9%	19.5%	1.6%
I have easy access to the materials I need to give advice on prudent antibiotic use and antibiotic resistance	25	128	104	31	15	78	3
	6.5%	33.3%	27.1%	8.1%	3.9%	20.3%	0.8%
I have good opportunities to provide advice on prudent antibiotic use to individuals	49	206	37	7	5	77	3
	12.8%	53.6%	9.6%	1.8%	1.3%	20.1%	0.8%
Motivation							
I know there is a connection between my dispensing of antibiotics and emergence and spread of antibiotic resistant bacteria	64	197	27	9	3	65	10
	16.7%	51.3%	7.0%	2.4%	0.8%	16.9%	2.6%
I have a key role in helping control antibiotic resistance	88	209	11	5	8	61	2
	22.9%	54.4%	2.9%	1.3%	2.1%	15.9%	0.5%

Abbreviations: SA: strongly agree; A: agree; D: disagree; SD: strongly disagree; N/A: not applicable; U: undecided; IDU: I do not understand.

**Table 4:** Awareness of initiatives that focus on antibiotic awareness and resistance.

Initiatives	No	%
TV or radio advertising for the public	70	18.2
Toolkits and resources for healthcare workers	70	18.2
Awareness raising from professional organizations	92	24.0
National or regional posters or leaflets on antibiotic awareness	58	15.1
Newspaper (national) articles on antibiotic resistance	33	8.6
National or regional guidelines on management of infections	56	14.6
Conference/Events focused on tackling antibiotic resistance	84	21.9
World Antibiotic Awareness Week	47	12.2
I am not aware of any initiatives	142	37.0

## Data Availability

All collected data for this study are published in this article.

## Supplementary Materials

The following supporting information can be downloaded at: https://www.mdpi.com/article/10.3390/antibiotics11020175/s1, Table S1: Behavior of pharmacists regarding prudent antimicrobial use, Table S2: Social media used by pharmacists for professional activities, Table S3: Details regarding information sources for management of infections, and Supplementary File S1: The study instrument.
