# Supplementary material for: Pharmacists’ Knowledge, Attitudes, Behaviors and Information Sources on Antibiotic Use and Resistance in Jordan
Source: Antibiotics (Basel). 2022 Jan 28;11(2):175. doi: 10.3390/antibiotics11020175 (PMC8868190; doi:10.3390/antibiotics11020175)
Supplement: Supplementary file 1 [file antibiotics-11-00175-s001.zip › antibiotics-1527786-Supplementary.pdf]

Table S1: Behavior of pharmacists regarding prudent antimicrobial use

Variable		N	%
How often did you dispense antibiotics during the last one week?	Once a day	29	7.6
	More than once a day	156	40.6
	Once a week	18	4.7
	More than once a week	70	18.2
	rarely	13	3.4
	never	29	7.6
	I do not remember	27	7
	Not Applicable	42	10.9
How often did you give out resources (e.g. leaflets or pamphlets) on prudent antibiotic use or management of infections to individuals during the last one week?	Once a day	16	4.2
	More than once a day	35	9.1
	Once a week	31	8.1
	More than once a week	30	7.8
	rarely	83	21.6
	never	98	25.5
	I do not remember	39	10.2
	Not Applicable	52	13.5
How often did you give out advice related to prudent antibiotic use or management of infections to an individual during the last one week?	Once a day	34	8.9
	More than once a day	105	27.3
	Once a week	55	14.3
	More than once a week	71	18.5
	rarely	40	10.4
	never	21	5.5
	I do not remember	29	7.6
	Not Applicable	29	7.6

Table S2: Social media used by pharmacists for professional activities

Which of the following social media networks do you mainly use for professional activities?	**N**	**%**
LinkedIn	45	11.7
YouTube	43	11.2
Facebook	309	80.5
Instagram	77	20.1
Google+	13	3.4
Pharmee app	1	0.3
What's up	5	1.3
Email	1	0.3
Google	71	18.5
Twitter	11	2.9
Telegram	3	0.8
I do not use social media	25	6.5

Table S3: Details regarding information sources for management of infections

Variable		N	%
If you were not able to give out advice or resources as frequently as you dispensed antibiotics, why was this?	Patient does not require information	53	13.8
	Difficulty getting patient to understand diagnosis	76	19.8
	Patient uninterested in information	174	45.3
	No resources available	142	37
	Insufficient time	104	27.1
	I was able to give out advice or resources as needed	99	25.8
	Language barriers	16	4.2
	I was not sure what advice to provide	20	5.2
	Not applicable	34	8.9
In the management of infections, which of these do you use regularly?	Clinical practice guidelines	138	35.9
	Documentation from the pharmaceutical industry	66	17.2
	Medical representatives from industry	54	14.1
	Continuing education training courses	234	60.9
	Previous clinical experience	112	29.2
	Infection specialists	30	7.8
	Professional resources/publications	109	28.4
	Social media	91	23.7
	Scientific journals	44	11.5
	I do not know	13	3.4
	None of the above	9	2.3
In the last 12 months, do you remember receiving any information about avoiding unnecessary dispensing of antibiotics?	No	136	35.4
	Unsure	29	7.6
	Yes	219	57%

Supplementary File S1: The study instrument.

Survey of pharmacists' knowledge and attitudes about antibiotics and antibiotic resistance

Dear Pharmacist,

You are invited to complete the following survey of pharmacists about their knowledge and attitudes about antibiotics and antibiotic resistance. Researchers from UK, Jordan University of Science and Technology, and Yarmouk University are seeking responses from pharmacists.
We would really value you completing the survey that will take 5 to 10 minutes to complete. It includes predominantly multiple-choice questions. Please feel free to cascade the link of the survey widely to colleagues.

In which language would you prefer to complete this survey?
English
Arabic

➢ Demographic Section

1. Please specify in which governorate you practice
Amman
Irbid
Salt
Zarqa
Madaba
Jerash
Ajloun
Mafraq
Karak
Tafilah
Ma'an
Aqaba

2. What is your predominant role? (i.e.>50% of your time)?
Generalist
Specialist
Academia/ Research

3. Where do you predominantly practice? (i.e. >50% of your time):
Hospital (any hospital type)
University (as an Academic) or research institute
Public clinic
Private clinic

4. How many years have you been practicing in your current profession?
0-2 years
3-5 years
6-10 years

11-15 years
16-20 years
21-25 years
>25 years

5. What is your age?
22-25 years
26-35 years
36-45 years
46-55 years
56-65 years
>66 years

6. What gender do you identify with?
Male
Female

➢ Knowledge about antibiotic use and antibiotic resistance

7. Please answer whether you believe these statements are true or false.

	True	False	Unsure
Antibiotics are effective against viruses			
Antibiotics are effective against cold infections			
Unnecessary use of antibiotics makes them become ineffective			
Taking antibiotics has associated side effects or risks such as diarrhea, colitis, allergies			
Every person treated with antibiotics is at an increased risk of antibiotic resistant infection			
Antibiotic resistant bacteria can spread from person to person			
Healthy people can carry antibiotic resistant bacteria			
The use of antibiotics to stimulate growth in farm animals is legal in Jordan			

❖ ***For the next questions, to what extent do you agree or disagree with the following statements:***

8. "I know what antibiotic resistance is"
Strongly disagree
Disagree
Undecided
Agree
Strongly Agree
I do not understand the question
Not Applicable

9. "I know there is a connection between my dispensing of antibiotics and emergence and spread of antibiotic resistant bacteria":
Strongly disagree
Disagree
Undecided
Agree
Strongly Agree
I do not understand the question

Not Applicable

10. "I know what information to give to individuals about prudent use of antibiotics and antibiotic resistance":

Strongly disagree
Disagree
Undecided
Agree
Strongly Agree
I do not understand the question
Not Applicable

11. "I have sufficient knowledge about how to use antibiotics appropriately for my current practice":

Strongly disagree
Disagree
Undecided
Agree
Strongly Agree
I do not understand the question
Not Applicable

12. "I have a key role in helping control antibiotic resistance":

Strongly Disagree
Disagree
Undecided
Agree
Strongly Agree
I do not understand the question
Not applicable

13. "I have easy access to guidelines I need on managing infections":

Strongly Disagree
Disagree
Undecided
Agree
Strongly Agree
I do not understand the question
Not applicable

14. "I have easy access to the materials I need to give advice on prudent antibiotic use and antibiotic resistance":

Strongly Disagree
Disagree
Undecided
Agree
Strongly Agree
I do not understand the question
Not applicable

15. "I have good opportunities to provide advice on prudent antibiotic use to individuals":

Strongly disagree
Disagree
Neutral

Agree
Strongly agree
I do not understand the question

16. "Environmental factors such as wastewater in the environment are important in contributing to antibiotic resistance in bacteria from humans"?

Strongly disagree
Disagree
Neutral
Agree
Strongly agree
I do not understand the question

17. "Excessive use of antibiotics in livestock and food production is important in contributing to antibiotic resistance in bacteria from humans"?

Strongly Disagree
Disagree
Undecided
Agree
Strongly Agree
I do not understand the question

- ***Please answer the following questions, considering the last one week only in your clinical practice:***

18. How often did you dispense antibiotics during the last one week?

Once a day
More than once a day
Once a week
More than once a week
rarely
never
I do not remember
Not Applicable

19. How often did you give out resources (e.g. leaflets or pamphlets) on prudent antibiotic use or management of infections to individuals during the last one week?

Once a day
More than once a day
Once a week
More than once a week
rarely
never
I do not remember
Not Applicable

20. How often did you give out advice related to prudent antibiotic use or management of infections to an individual during the last one week?

Once a day
More than once a day
Once a week
More than once a week
Rarely
Never

I do not remember

21. If you were not able to give out advice or resources as frequently as you dispensed antibiotics, why was this?
Patient does not require information
Difficulty getting patient to understand diagnosis
Patient uninterested in information
No resources available
Insufficient time
I was able to give out advice or resources as needed
Language barriers
I was not sure what advice to provide
Not applicable

➢ Sources of information about avoiding unnecessary dispensing of antibiotics Section

22. Which of the following social media networks do you mainly use for professional activities?
(Choose all that apply)
Twitter
Facebook
LinkedIn
Google+
YouTube
Instagram
I do not use social media
Others_____________________________________

23. In the management of infections, which of these do you use regularly? (Choose all that apply)
Clinical practice guidelines
Documentation from the pharmaceutical industry
Medical representatives from industry
Previous clinical experience
Continuing education training courses
Infection specialists
Scientific journals
Professional resources/publications
Social media
None of the above
I do not know
Others: ____

24. In the last 12 months, do you remember receiving any information about avoiding unnecessary dispensing of antibiotics?
Yes
No
Unsure

25. How did you first get this information about avoiding unnecessary dispensing of antibiotics? (Select all that apply).
Colleague or peer

My workplace
Media (TV/Radio) adverts
Social Media
Newspaper
Published guidelines
Training - conference/group
Training - one to one
Government policy
Scientific organization
My medical professional body
Audit and feedback
Others: ____

26. Did the information contribute to changing your views about avoiding unnecessary dispensing of antibiotics?
Yes
No
Unsure

27. Which source(s) of information has had the most influence on changing your views? Select no more than 2.
Colleague or peer
My workplace
Media (TV/radio) adverts
Social media
Newspaper
Published guidelines
Training - conference/group
Training - one to one
Government policy
Scientific organization
My medical professional body
Audit and feedback
Others: ____

28. On the basis of the information you received, have you changed your practice on dispensing of antibiotics?
Yes
No
Unsure

➢ Awareness of initiatives and national action plans on antimicrobial resistance Section

29. What initiatives are you aware of which focus on antibiotic awareness and resistance? Select all that apply
TV or Radio advertising for the public
Toolkits and resources for healthcare workers
National or regional guidelines on management of infections
Awareness raising from professional organizations
Conference/Events focused on tackling antibiotic resistance
National or regional posters or leaflets on antibiotic awareness
Newspaper (national) articles on antibiotic resistance
World Antibiotic Awareness Week
I am not aware of any initiatives

30. Does your country have a national action plan on antimicrobial resistance?

Yes
No
Unsure

31. On which topics would you like to receive more information? (Choose all that apply)

Resistance to antibiotics
How to use antibiotics
Medical conditions for which antibiotics are used
Prescription of antibiotics
Links between the health of humans, animals and the environment
None
Others: ___________________________________
